# Sorafenib/piroxicam therapy with biomarker-defined survival benefit in canine muscle-invasive urothelial carcinoma

**DOI:** 10.1038/s41598-026-64955-1

**Published:** 2026-09-01

**Authors:** Shingo Maeda, Shohei Yokota, Mao Komori, Yuko Goto-Koshino, James K. Chambers, Kazuyuki Uchida, Tomohiro Yonezawa, Yasuyuki Momoi

**Affiliations:** 1 Laboratory of Veterinary Clinical Pathobiology, Graduate School of Agricultural and Life Sciences, The University of Tokyo, 1-1-1, Yayoi, Bunkyo-ku, Tokyo, 113-8657 Japan; 2 Molecular Diagnostic Laboratory, Veterinary Medical Center, The University of Tokyo, Tokyo, Japan; 3 Laboratory of Veterinary Pathology, Graduate School of Agricultural and Life Sciences, The University of Tokyo, Tokyo, Japan

**Keywords:** Bladder cancer, Canine, On-target toxicity, Spontaneous animal model, Transitional cell carcinoma, Biomarkers, Cancer, Oncology, Urology

## Abstract

Molecular-targeted therapies require biomarker-driven evaluation to optimize precision oncology strategies; however, translational models integrating clinical outcomes with mechanistic biomarkers are limited. Canine urothelial carcinoma shares molecular features with human muscle-invasive bladder cancer, including activation of VEGFR signaling, providing a valuable comparative oncology platform. Sorafenib is a multi-kinase inhibitor targeting VEGFRs, PDGFRβ, BRAF, and related pathways, but its therapeutic mechanisms and clinical benefit in urothelial carcinoma remain incompletely understood. In this study, 43 dogs with naturally occurring urothelial carcinoma received first-line sorafenib and piroxicam therapy. The overall response rate was 62.8%, with median progression-free and overall survival of 175 (range, 31–665) and 407 (range, 103–1,723) days, respectively. The results exceeded outcomes in historical controls treated with piroxicam alone. Treatment was generally well tolerated, with only three grade 3 adverse events and no grade 4–5 toxicities. Biomarker analyses revealed that treatment-induced hypertension and high tumor *CX3CL1* mRNA expression were independently associated with improved overall survival, suggesting that effective VEGFR pathway inhibition and modulation of the tumor immune microenvironment contribute to clinical benefit. These findings support sorafenib-based therapy as a promising strategy for canine urothelial carcinoma and highlight the translational potential of biomarker-driven VEGFR-targeted approaches in precision oncology.

**Supplementary Information:**

The online version contains supplementary material available at https://doi.org/10.1038/s41598-026-64955-1.

## Introduction

Bladder cancer, also known as urothelial carcinoma, is a common, aggressive malignancy in humans that accounts for approximately 0.6 million new patients and nearly 200,000 deaths worldwide each year^1^. Histologically, urothelial carcinoma is classified as either low-grade superficial or high-grade muscle-invasive types^2^. Despite platinum-based chemotherapy as the standard first-line treatment, advanced urothelial carcinoma has a poor prognosis, with a median overall survival of 9–15 months and a 5-year survival rate of 5% for metastatic disease^3,4^. A substantial proportion of patients are ineligible for cisplatin-based chemotherapy owing to renal dysfunction or other comorbidities, which underscores the urgent need for alternative therapeutic strategies.

Sorafenib is a multi-kinase inhibitor that targets Raf serine/threonine kinases (e.g., BRAF and CRAF), vascular endothelial growth factor receptors (VEGFRs), platelet-derived growth factor receptor beta (PDGFRβ), and KIT, and thereby suppresses both oncogenic signaling and tumor angiogenesis^5^. Sorafenib received approval for use in the treatment of several human malignancies, including hepatocellular, renal, and thyroid carcinomas. In preclinical studies, sorafenib exerted antitumor effects in bladder cancer models by inhibiting the Raf/MEK/ERK pathway and angiogenic signaling^6–8^. However, subsequent phase I and II clinical trials in patients with muscle-invasive or metastatic urothelial carcinoma demonstrated limited efficacy^9–11^, and the extent of the clinical benefit remains unclear.

Naturally occurring urothelial carcinoma in dogs closely resembles human bladder cancer in terms of genetic and environmental heterogeneity, clinical signs, histopathological features, metastatic behavior, and response to chemotherapy, particularly cisplatin-based regimens^12–14^. Unlike humans, the high-grade muscle-invasive type of urothelial carcinoma predominates in dogs, and accounts for more than 90% of the cases. Gene expression profiling using microarrays and RNA sequencing revealed remarkable similarities between canine and human urothelial carcinoma^15–18^, which underscores the translational value of the canine model. Furthermore, clinical trials in dogs can be completed in a relatively short period owing to their shorter life expectancy than that of humans. Therefore, naturally occurring urothelial carcinoma in dogs is a valuable, efficient model for evaluating therapeutic strategies in humans.

A somatic point mutation in the BRAF gene (BRAF^V595E^), which is homologous to the human BRAF^V600E^ mutation, is present in more than 70% of dogs with urothelial carcinoma^19^. A previous RNA sequencing study showed that the BRAF^V595E^ mutation was associated with the upregulation of genes involved in tissue development, cell cycle, and cell death, and the downregulation of genes involved in the immune response^18^. Furthermore, VEGFR2 and PDGFRβ, which constitute the target molecules of sorafenib, were highly expressed in tumor tissues of most cases of canine urothelial carcinoma^20,21^. The antitumor effect of sorafenib has been demonstrated in canine urothelial carcinoma cell lines and a xenograft mouse model^22,23^. These findings suggest that a therapeutic approach targeting BRAF- and angiogenesis-related pathways using sorafenib may be rational for treating canine urothelial carcinoma.

In the present study, we conducted a prospective single-arm clinical trial to evaluate the efficacy and safety of sorafenib in combination with the nonsteroidal anti-inflammatory drug (NSAID) piroxicam as a first-line treatment for dogs with naturally occurring muscle-invasive urothelial carcinoma. Furthermore, we aimed to identify biomarker-defined predictors of therapeutic response and survival, integrating molecular, immunological, and treatment-related parameters to advance precision oncology through a comparative oncology framework.

## Results

### Case population

Between November 2019 and December 2021, 55 dogs were screened for inclusion in the study at the Veterinary Medical Center of the University of Tokyo (VMC-UT). Of these, 12 were excluded owing to withdrawal of consent (*n* = 4), hydronephrosis caused by tumor obstruction (*n* = 4), end-stage urothelial carcinoma (*n* = 2), end-stage kidney disease (*n* = 1), or complete urethral obstruction caused by tumor invasion (*n* = 1). Therefore, a total of 43 dogs with urothelial carcinoma were enrolled in this study. The case dogs included 28 females (1 intact and 27 spayed) and 15 males (1 intact and 14 castrated), and the median age was 12.1 (range, 7.6–17.1) years. According to the World Health Organization (WHO) TNM classification for canine bladder cancer^24,25^, 35 of 43 (81%) tumors were classified as T2 (muscle-invasive), and 8 of 43 (19%) as T3 (tumor-invading neighboring organs). None of the tumors were classified as T1 (superficial papillary tumors). Nodal and distant (to the lungs) metastases were observed in 10 (23%) and 9 (21%) dogs, respectively. The baseline characteristics of the dogs included in this clinical trial are summarized in Table 1 and Supplementary Table S1.

**Table 1:** Baseline subject characteristics in dogs treated with sorafenib/piroxicam (*n* = 43).

Characteristics	No. of dogs (%)
Age (years)	
Median	12.1
Range	7.6–17.1
Body weight (kg)	
Median	6.9
Range	2.7–29.5
Sex	
Female neutered	27 (62.8)
Female intact	1 (2.3)
Male neutered	14 (32.6)
Male intact	1 (2.3)
Tumor volume (mm^3^)	
Median	2,834
Range	350–25,833
Tumor location	
Vesical apex	5 (11.6)
Vesical body	11 (25.6)
Vesical trigone	25 (58.1)
Urethra involvement	21 (48.8)
Prostate involvement	5 (11.6)
Muscle-invasive tumor (T2 or T3)	
Yes	43 (100)
No	0 (0)
Metastasis	
Any	18 (41.9)
Lymph node	10 (23.3)
Lung	9 (20.9)
BRAF gene mutation	
Wild-type	16 (37.2)
BRAF^V595E^ mutation	27 (62.8)

### Efficacy

**Fig. 1:**
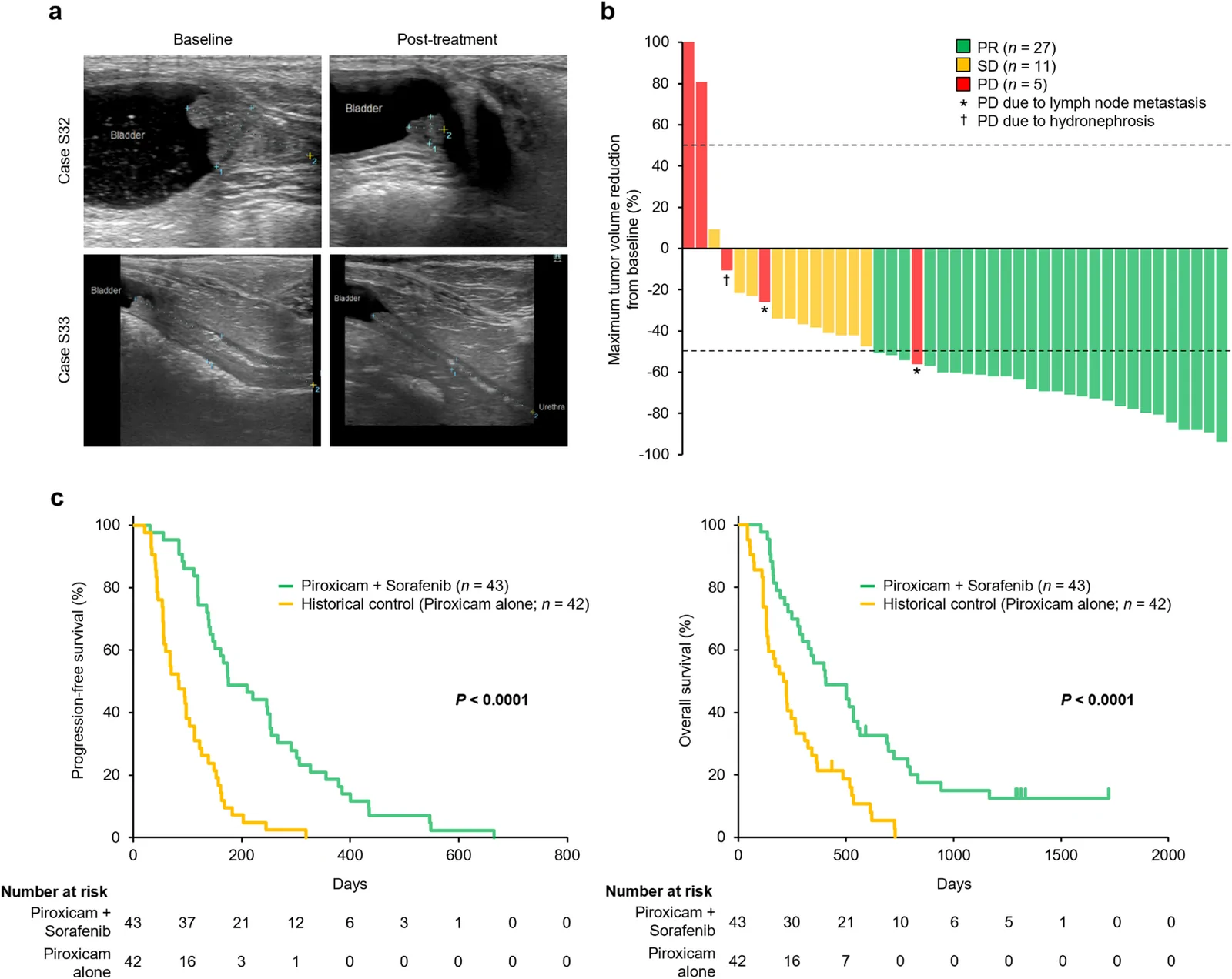
Sorafenib induces clinical response and improves survival in dogs with urothelial carcinoma. (**a**) Representative ultrasonographic images of bladder and urethral masses in dogs treated with sorafenib and piroxicam. In case S32, the bladder mass shrunk 8 weeks after treatment compared to baseline. In case S33, the urethral tumor thinned out 4 weeks after treatment. (**b**) Waterfall plot showing the maximum percentage of tumor-burden reduction from baseline in dogs treated with sorafenib and piroxicam (*n* = 43). Dashed lines indicate ± 50%. Asterisk and dagger indicate progressive disease owing to lymph node metastasis and hydronephrosis, respectively. PR, partial response; SD, stable disease; PD, progressive disease. (**c**) Progression-free survival (left) and overall survival (right) in dogs treated with sorafenib and piroxicam (*n* = 43, green line) or in the historical control (dogs treated with piroxicam alone; *n* = 42, yellow line). Log-rank test.

The response data are presented in Supplementary Table S1. Combination treatment with sorafenib and piroxicam reduced the tumor burden in more than half of the dogs with urothelial carcinoma after 4–8 weeks (Fig. 1a and Supplementary Movie 1). The best percentage change in the target lesions from baseline for each dog is shown in Fig. 1b. Among the 43 dogs treated with sorafenib/piroxicam, 27 (62.8%) had a partial response (PR), 11 (25.6%) had stable disease (SD), and five (11.6%) had progressive disease (PD). At the cutoff time for the study data (May 31, 2025), 6 dogs (14.0%) were still alive. The median progression-free survival (PFS) and overall survival (OS) in dogs treated with sorafenib/piroxicam were 175 (range, 31–665) and 407 (range 103–1,723) days, respectively. A comparison of the baseline clinical characteristics between the sorafenib/piroxicam cohort and the historical control cohort treated with piroxicam alone^26^ is provided in Supplementary Table S2. No significant differences were identified in any baseline clinical characteristics between the two cohorts. The PFS and OS of dogs treated with sorafenib in combination with piroxicam were longer than those of dogs treated with piroxicam alone (Fig. 1c). The median duration of sorafenib/piroxicam treatment was 295 (range 31–1,723) days. Sorafenib and piroxicam treatment was continued for more than 6 months in 27/43 (62.8%) dogs in this study.

### Adverse events

Of the 43 dogs that received sorafenib and piroxicam, 36 (84%) experienced adverse effects (Table 2). Seven dogs experienced no adverse events. The majority of treatment-related adverse events were grade 1 or 2 and many were transient. The leading adverse events were systemic hypertension (49%), increased alanine aminotransferase levels (35%), increased alkaline phosphatase levels (16%), diarrhea (16%), and increased creatinine levels (14%). One dog (2%) developed grade 3 acute kidney injury and was treated with fluid therapy and piroxicam withdrawal. Dermatologic adverse events were observed in seven cases (16%), including hyperpigmentation, pruritus, and alopecia. There were no grade 4–5 treatment-related adverse events. None of the dogs experienced an event that led to sorafenib discontinuation or dose reduction.

**Table 2:** Adverse events in dogs treated with sorafenib/piroxicam (*n* = 43).

Event^a^	Number of cases (%)		
	Grade 1	Grade 2	Grade 3
Any event	28 (65.1)	18 (41.9)	3 (7.0)
Gastrointestinal/pancreas			
Vomiting	2 (4.7)	2 (4.7)	0
Diarrhea	6 (14.0)	1 (2.3)	0
Anorexia	2 (4.7)	2 (4.7)	0
Pancreatitis	0	2 (4.7)	0
Dermatologic			
Hyperpigmentation	0	2 (4.7)	0
Pruritus	2 (4.7)	0	0
Alopecia	3 (7.0)	0	0
Cardiac			
Systemic hypertension	9 (20.9)	9 (20.9)	3 (7.0)
Neurologic			
Hypertensive encephalopathy	0	0	1 (2.3)
Renal			
Acute kidney injury	0	0	1 (2.3)
Metabolic^b^			
Increased ALT	12 (27.9)	3 (7.0)	0
Increased ALP	7 (16.3)	0	0
Increased total bilirubin	1 (2.3)	0	0
Increased creatinine	4 (9.3)	2 (4.7)	0

^a^Toxicity grade based on published criteria^50^.

^b^ALT, alanine aminotransferase; ALP, alkaline phosphatase.

**Fig. 2:**
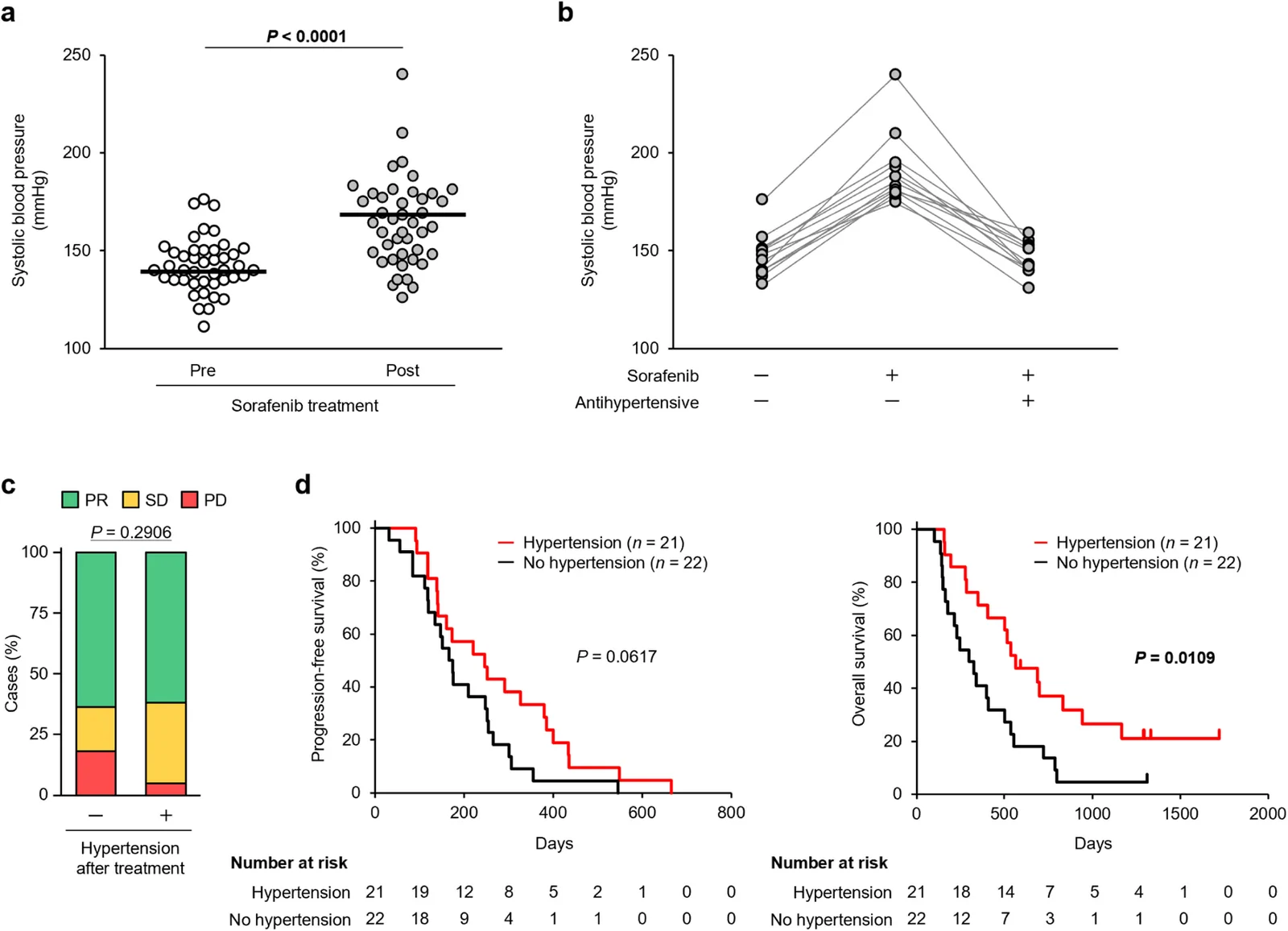
Posttreatment hypertension is associated with survival in dogs treated with sorafenib and piroxicam. (**a**) Systolic blood pressure before and after sorafenib treatment (*n* = 43). Median values are depicted by horizontal lines. The Mann–Whitney *U* test. (**b**) The effect of antihypertensive agents on dogs with sorafenib-induced hypertension (Grade 2 or 3; *n* = 12). (**c**) Association of hypertension after sorafenib treatment with clinical response (*n* = 43). Cases were classified as either hypertension (Grade 1–3; *n* = 21) or no hypertension (*n* = 22). PR, partial response; SD, stable disease; PD, progressive disease. Cochran–Armitage test. (**d**) Kaplan–Meier curves of progression-free survival (left) and overall survival (right) according to the presence of posttreatment hypertension in dogs treated with sorafenib and piroxicam (*n* = 43). Log-rank test.

Systemic hypertension was the most common adverse event (Table 2). The systolic blood pressure (SBP) following sorafenib treatment was higher than the baseline level (Fig. 2a). A post-treatment SBP greater than 170 mmHg was observed in 12 dogs (28%), including grades 2 and 3 systemic hypertension in nine (21%) and three (7%) cases, respectively. The dogs were treated with antihypertensive drugs, such as amlodipine and telmisartan. After receiving therapy, SBP normalized in all cases (Fig. 2b). In addition, one dog with grade 3 systemic hypertension exhibited grade 3 hypertensive encephalopathy, which was effectively controlled with amlodipine.

### Sorafenib dose and clinical outcomes

The median dose of sorafenib administered was 7.3 mg/kg (range, 4.6–10.1 mg/kg; Supplementary Table S1). To evaluate the impact of sorafenib dosage, cases were divided into two groups based on the median dose. Sorafenib dose was not significantly associated with treatment response, although a trend toward improved response with higher doses was observed (Supplementary Fig. S1a). There was also no significant difference between the high- and low-dose groups in either PFS or OS (Supplementary Fig. S1b). In addition, sorafenib dose was not significantly associated with the occurrence of adverse events (Supplementary Table S3) or treatment-associated hypertension (Supplementary Table S4).

### Hypertension and clinical outcomes

In humans, the effects of sorafenib are associated with posttreatment hypertension^27,28^. Therefore, we examined the association between hypertension following sorafenib treatment and the outcomes in this canine clinical trial. Of the 43 dogs that received sorafenib treatment, hypertension (grades 1–3) was observed in 21 (Table 2 and Supplementary Table S1). There was no significant association between the development of hypertension and response to treatment (Fig. 2c). The OS was significantly longer in dogs that developed hypertension after treatment than in those that did not (Fig. 2d). Although not statistically significant, the PFS tended to be longer in the hypertensive group than in the non-hypertensive group (Fig. 2d).

### Biomarker assessments

Sorafenib exerts antitumor effects via multiple targets, including Raf serine/threonine kinases (e.g., BRAF), VEGFRs, and PDGFR-β^5^. Previously, we demonstrated that VEGFR2 signaling in canine urothelial carcinoma cells induces CX3CL1 expression, which leads to tumor progression by recruiting myeloid-derived suppressor cells (MDSCs), such as polymorphonuclear MDSCs (PMN-MDSCs) and monocytic MDSCs (M-MDSCs). Sorafenib inhibits this pathway and exerts antitumor effects^22,23^. Therefore, this study evaluated the associations between clinical outcomes and related molecular markers, including BRAF^V595E^ mutation status, tumor and urinary levels of VEGF-A, PDGF-BB, and CX3CL1, as well as circulating PMN-MDSCs and M-MDSCs.

**Fig. 3:**
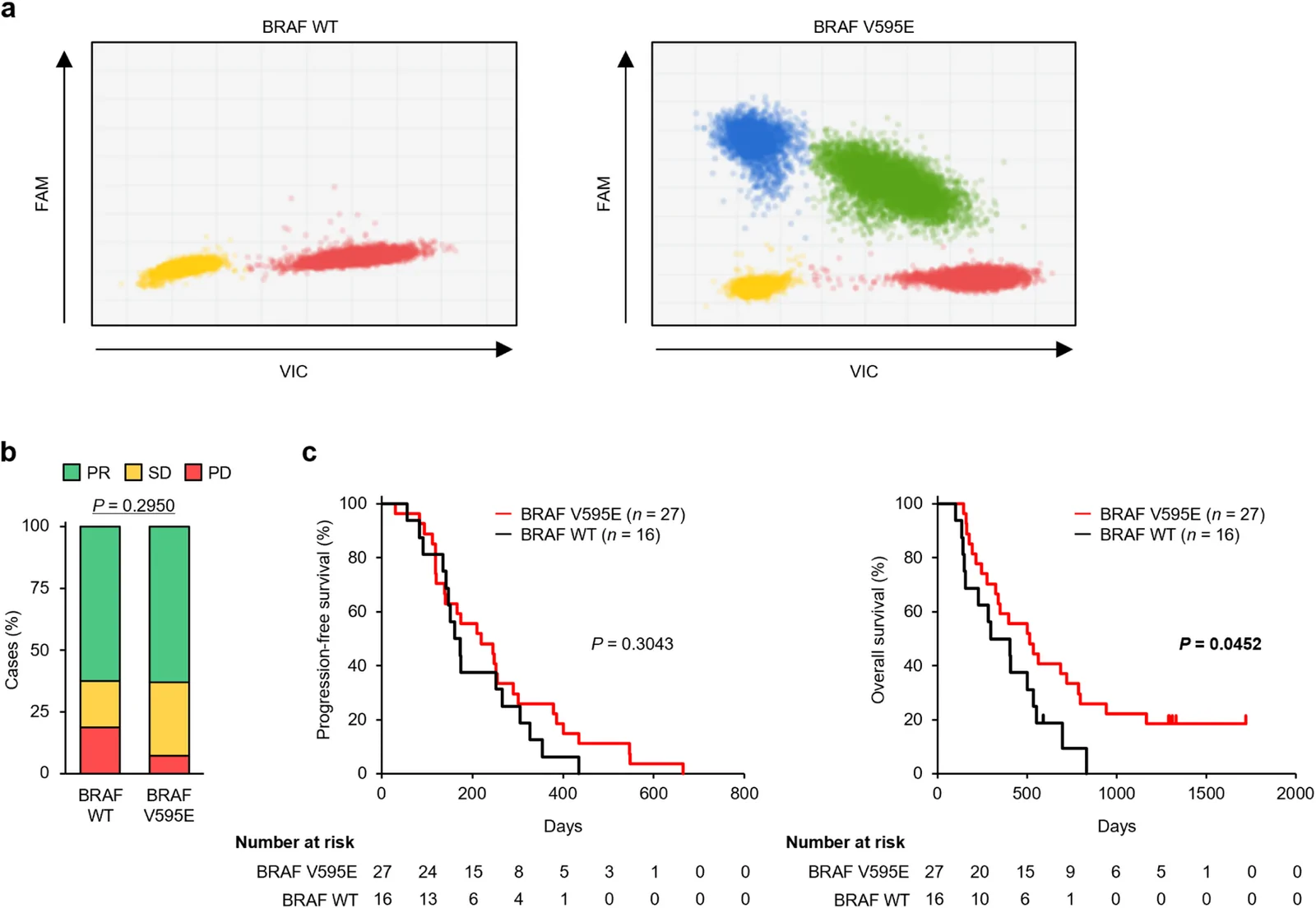
BRAF^V595E^ mutation is associated with survival in dogs treated with sorafenib and piroxicam. (**a**) Digital PCR assay for genotyping of BRAF in dogs with urothelial carcinoma. X-axis: VIC, wild-type (WT) BRAF. Y-axis: FAM, BRAF^V595E^ mutation. Absence of blue and green clusters indicates urothelial carcinoma with wild-type BRAF (left). Presence of blue and green clusters indicates urothelial carcinoma with BRAF^V595E^ mutation (right). (**b**) Clinical responses in canine urothelial carcinoma cases (*n* = 43) with wild-type BRAF (*n* = 16) or BRAF^V595E^ mutation (*n* = 27). PR, partial response; SD, stable disease; PD, progressive disease. Cochran–Armitage test. (**c**) Kaplan–Meier curves of progression-free survival (left) and overall survival (right) according to BRAF gene status in dogs treated with sorafenib and piroxicam (*n* = 43). Log-rank test.

The BRAF^V595E^ mutation was detected in 27 of the 43 cases (Table 1; Fig. 3a). Among the 27 cases positive for the BRAF^V595E^ mutation, the median variant allele frequency (VAF) was 35.2% (range, 3.1%–56.2%; Supplementary Table S1). There was no association between the BRAF^V595E^ mutation and clinical response in dogs treated with sorafenib and piroxicam (Fig. 3b). The BRAF^V595E^ mutation was associated with OS, but not PFS (Fig. 3c).

**Fig. 4:**
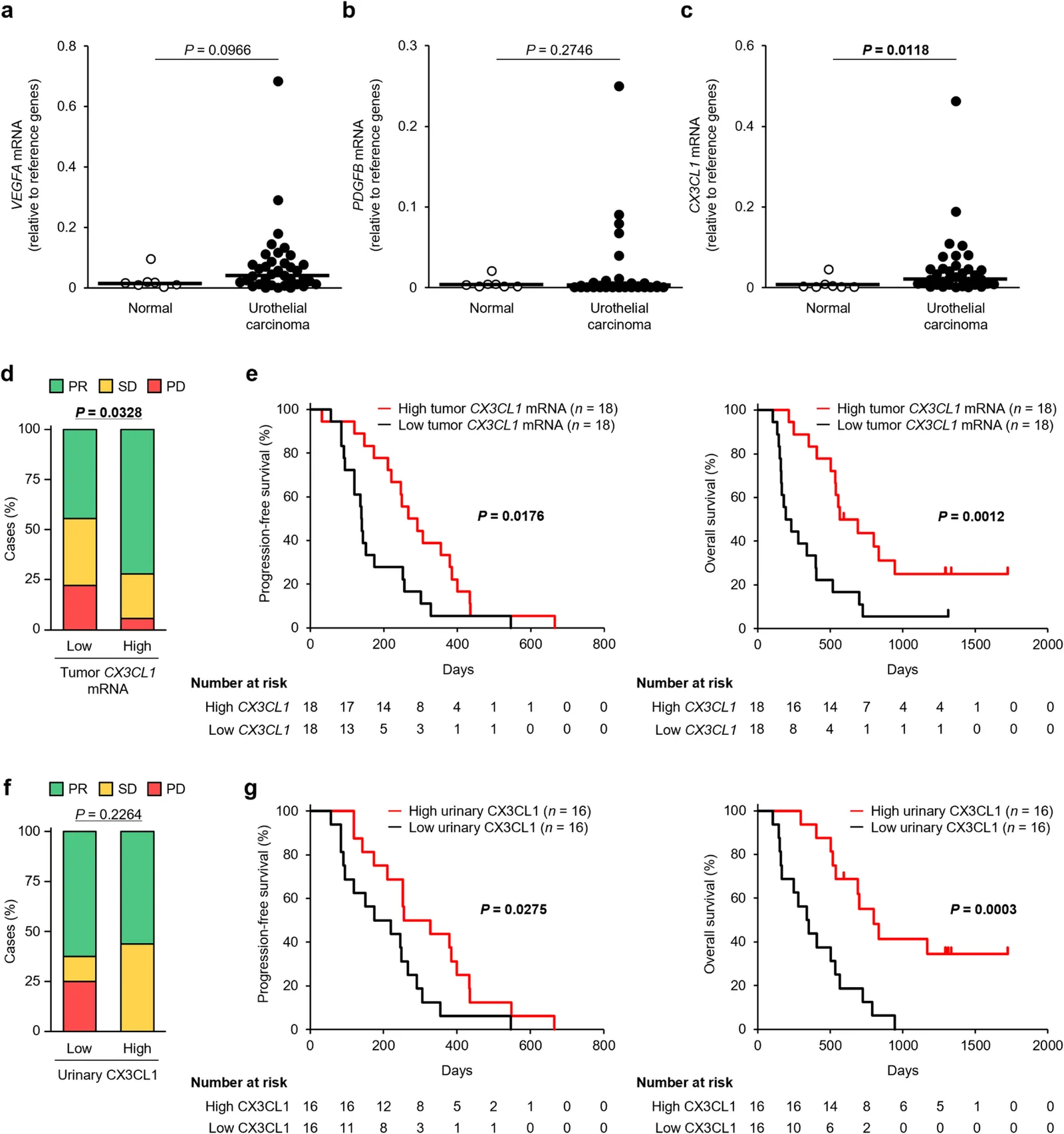
Association of molecular markers with clinical responses and survival in dogs treated with sorafenib and piroxicam. (**a**–**c**) Expression of *VEGFA* (**a**), *PDGFB* (**b**), and *CX3CL1* (**c**) mRNA in the bladder of normal dogs (*n* = 7) and dogs with urothelial carcinoma (*n* = 36). Gene expression levels are normalized to the reference genes and presented as relative values. Median values are depicted by horizontal lines. The Mann–Whitney *U* test. (**d**) Association of baseline tumor *CX3CL1* mRNA levels with clinical response (*n* = 36). Cases were classified as either high or low tumor *CX3CL1* levels (*n* = 18 each) according to the median. PR, partial response; SD, stable disease; PD, progressive disease. Cochran–Armitage test. (**e**) Kaplan–Meier curves of progression-free survival (left) and overall survival (right) according to baseline tumor *CX3CL1* mRNA levels in dogs treated with sorafenib and piroxicam (*n* = 36). Cases were classified as either high or low tumor *CX3CL1* levels (*n* = 18 each) according to the median. Log-rank test. (**f**) Association of baseline urinary CX3CL1 levels with clinical response (*n* = 32). Cases were classified as either high or low urinary CX3CL1 levels (*n* = 16 each) according to the median. PR, partial response; SD, stable disease; PD, progressive disease. Cochran–Armitage test. (**g**) Kaplan–Meier curves of progression-free survival (left) and overall survival (right) according to baseline urinary CX3CL1 levels in dogs treated with sorafenib and piroxicam (*n* = 32). Cases were classified as either high or low urinary CX3CL1 levels (*n* = 16 each) according to the median. Log-rank test.

Of the 43 enrolled cases, tumor expression of *VEGFA*, *PDGFB*, and *CX3CL1* mRNA was evaluated in 36 dogs via quantitative RT-PCR. Although tumor *VEGFA* and *PDGFB* levels tended to be higher in the affected dogs, the differences between the two groups were not statistically significant (Fig. 4a, b). Tumor *CX3CL1* mRNA levels were higher in dogs with urothelial carcinoma than in healthy controls (Fig. 4c). Cases were subsequently classified into high or low expression groups based on median values. High tumor *CX3CL1* expression was associated with a favorable clinical response in dogs treated with sorafenib/piroxicam (Fig. 4d). Similarly, both PFS and OS were significantly longer in the high *CX3CL1* group than in the low group (Fig. 4e). In contrast, tumor *VEGFA* and *PDGFB* mRNA levels showed no association with clinical response, PFS, or OS (Supplementary Figs. S2 and S3).

The urinary levels of CX3CL1, VEGF-A, and PDGF-BB were higher in dogs with urothelial carcinoma than in healthy dogs^22^. Of the 43 dogs in this trial, the urinary levels of CX3CL1, VEGF-A, and PDGF-BB were measured in 32, 30, and 30 dogs, respectively. The median urinary levels were 1,615 pg/mg creatinine (range, 483–6,357) for CX3CL1, 214 pg/mg creatinine (range, 72–974) for VEGF-A, and 15 pg/mg creatinine (range, 0–167) for PDGF-BB. Based on these median levels, each case was classified into either the high or low group. There was no significant association between urinary CX3CL1 levels and the clinical response in dogs treated with sorafenib/piroxicam (Fig. 4f). However, PFS and OS were longer in dogs with high urinary CX3CL1 than in those with low levels (Fig. 4g). In contrast, urinary VEGF-A and PDGF-BB levels were not associated with clinical response, PFS, or OS (Supplementary Figs. S2 and S3).

**Fig. 5:**
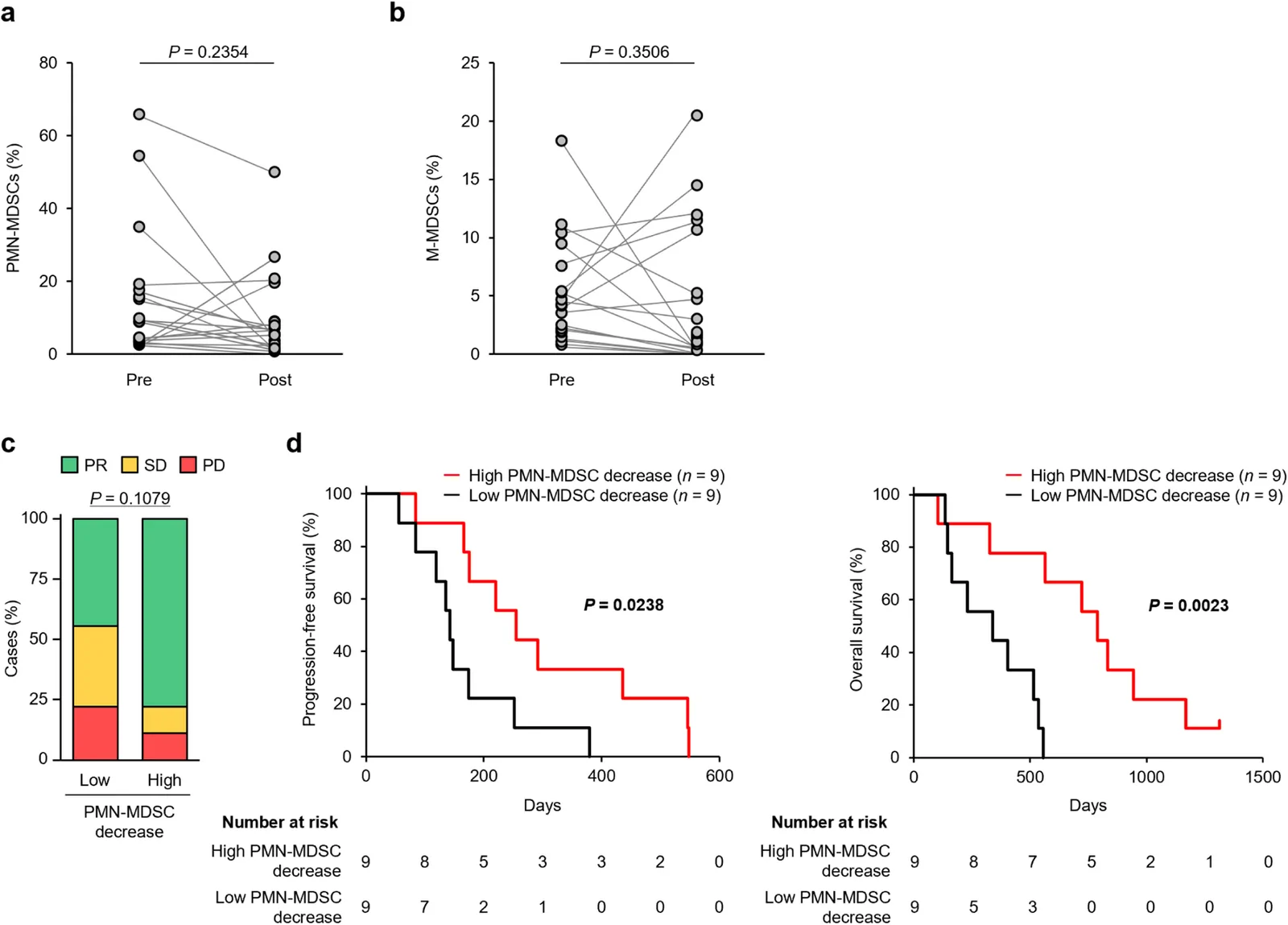
Posttreatment reduction in circulating myeloid-derived suppressor cells (MDSCs) is associated with survival in dogs treated with sorafenib and piroxicam. (**a**) Proportions of polymorphonuclear MDSCs (PMN-MDSCs) before and after sorafenib treatment (*n* = 18). The Mann–Whitney *U* test. (**b**) Proportions of monocytic MDSCs (M-MDSCs) before and after sorafenib treatment (*n* = 18). The Mann–Whitney *U* test. (**c**) Association between the decrease in the ratio of PMN-MDSCs after sorafenib treatment and clinical response (*n* = 18). Cases were classified as either a high or low decrease in PMN-MDSCs (*n* = 9 each) according to the median. PR, partial response; SD, stable disease; PD, progressive disease. Cochran–Armitage test. (**d**) Kaplan–Meier curves of progression-free survival (left) and overall survival (right) according to the posttreatment reduction in PMN-MDSC in dogs treated with sorafenib and piroxicam (*n* = 18). Cases were classified as either a high or low decrease in PMN-MDSCs (*n* = 9 each) according to the median. Log-rank test.

Circulating PMN-MDSCs and M-MDSCs were increased in dogs with urothelial carcinoma and were associated with advanced tumor stage^29^. Moreover, sorafenib treatment decreased the number of MDSCs in the tumor tissue of canine urothelial carcinoma-engrafted mice^22^. This trial evaluated circulating PMN-MDSCs and M-MDSCs in 18 of 43 dogs before and after sorafenib treatment. The median baseline proportions of PMN-MDSCs and M-MDSCs were 8.8% (range 2.5–65.8%) and 4.2% (range 0.8–18.3%), respectively. After sorafenib administration, decreases in PMN-MDSCs and M-MDSCs were observed in 12 dogs, whereas 6 dogs showed an increase (Fig. 5a, b). The median rates of decrease in PMN-MDSCs and M-MDSCs following sorafenib treatment were − 34.4% (range − 95.8% to 656.4%) and − 31.5% (range − 89.8% to 338.5%), respectively. Dogs were assigned to the high and low groups based on the median baseline values of PMN-MDSCs and M-MDSCs as well as the median values of their respective posttreatment reduction rates. Although the degree of PMN-MDSC reduction tended to correlate with the clinical response, it was not statistically significant (Fig. 5c). PFS and OS were longer in dogs with a greater reduction in PMN-MDSCs than in those with a smaller reduction (Fig. 5d). Baseline levels of PMN-MDSCs and M-MDSCs, as well as the M-MDSC reduction rate after sorafenib administration were not significantly associated with the clinical response, PFS, or OS (Supplementary Fig. S4).

### Multivariate survival analyses

To identify independent prognostic factors under sorafenib treatment, univariate and multivariate Cox proportional hazards analyses were performed. Variables that showed statistical significance in the univariate analyses (Supplementary Table S5) were considered for inclusion in the multivariable models. Age, BRAF^V595E^ mutation status, posttreatment hypertension, and tumor *CX3CL1* mRNA expression were selected for inclusion in the multivariate Cox proportional hazards models based on statistical significance in the univariate analyses and biological relevance. Although the reduction rate of circulating PMN-MDSCs was associated with PFS and OS in univariate analyses, this parameter was only available for 18 of the 43 dogs because paired pre- and post-treatment samples were required. Thus, it was not included in the multivariable analyses to preserve statistical robustness. In addition, urinary CX3CL1 levels were also associated with survival in univariate analyses. However, urinary CX3CL1 levels were correlated with tumor *CX3CL1* expression. Therefore, tumor *CX3CL1* mRNA expression was selected for inclusion in the multivariate models to avoid potential collinearity and to prioritize a variable that more directly reflects tumor-derived expression.

In the multivariable analysis for PFS, none of the included variables reached statistical significance, although high tumor *CX3CL1* mRNA expression showed a trend toward prolonged PFS (Table 3). The multivariable analysis for OS identified age ≥ 12 years as an independent predictor of poor prognosis (HR, 2.52; 95% CI, 1.00–6.32; *P* = 0.049). Posttreatment hypertension (HR, 0.41; 95% CI, 0.18–0.92; *P* = 0.031) and high tumor *CX3CL1* mRNA expression (HR, 0.39; 95% CI, 0.18–0.84; *P* = 0.016) were independently associated with improved OS (Table 3). BRAF^V595E^ mutation was not significantly associated with OS after adjustment for covariates (HR, 0.69; 95% CI, 0.29–1.61; *P* = 0.384).

**Table 3:** Multivariate analysis for survival with Cox proportional hazards model.

Characteristics	Progression-free survival		Overall survival	
	HR (95% CI)	*P* value	HR (95% CI)	*P* value
Age				
<12 years	Reference		Reference	
≥12 years	1.17 (0.52–2.59)	0.707	2.52 (1.00–6.32)	**0.049**
BRAF gene mutation				
Wild-type	Reference		Reference	
BRAF^V595E^ mutation	0.76 (0.34–1.71)	0.505	0.69 (0.29–1.61)	0.384
Posttreatment hypertension				
No	Reference		Reference	
Yes	0.79 (0.38–1.65)	0.539	0.41 (0.18–0.92)	**0.031**
Tumor *CX3CL1* mRNA				
Low	Reference		Reference	
High	0.48 (0.23–1.01)	0.052	0.39 (0.18–0.84)	**0.016**

HR, hazard ratio; CI, confidence Interval.

## Discussion

In the present study, we demonstrated that first-line sorafenib in combination with piroxicam provided clinical benefits to dogs with naturally occurring muscle-invasive urothelial carcinoma. The objective response rate (ORR) of sorafenib/piroxicam treatment was 63% (27 of 43 dogs), with a median PFS and median OS of 175 and 407 days, respectively. Although a randomized clinical trial would be needed to establish the significance of the observed differences, the efficacy of sorafenib treatment compares favorably with that reported for conventional first-line treatments in canine urothelial carcinoma, including piroxicam monotherapy (ORRs 12–21%; median PFS range 90–120 days; median OS range 216–244 days), cisplatin-based regimens (ORRs 11–73%; median PFS range 78–186 days; median OS range 179–329 days), and mitoxantrone in combination with piroxicam (ORR 35%; median PFS 194 days; median OS 350 days)^14,26,30–33^. The median OS range of dogs with urothelial carcinoma treated with surgery followed by adjuvant therapy is 348–385 days^34,35^, suggesting that sorafenib in combination with piroxicam achieves survival outcomes that are at least comparable to, and potentially exceed, those obtained with conventional therapy. Recent studies have shown that several molecular targeted therapies are effective in treating canine urothelial carcinoma. The BRAF inhibitor vemurafenib exerts antitumor activity in dogs with urothelial carcinoma that harbor the BRAF^V595E^ mutation (ORR 38%; median PFS 181 days; median OS 354 days)^36^. The clinical benefits of treating dogs with urothelial carcinoma with the HER2/EGFR dual inhibitor lapatinib (ORRs 55%; median PFS 193 days; median OS 435 days) or the anti-CCR4 antibody mogamulizumab (ORRs 53–71%; median PFS range 228–241 days; median OS range 468–474 days) have been reported^26,37,38^. In this study, sorafenib treatment for canine urothelial carcinoma had therapeutic effects equivalent to those of other molecular-targeted drugs reported to date.

Sorafenib/piroxicam treatment was generally well-tolerated. Most adverse events were mild to moderate and manageable. There were no grade 4 or 5 toxicities; moreover, treatment discontinuation or dose reduction of sorafenib owing to adverse events was not necessary, which underscores the feasibility of this regimen as a first-line therapy in clinical practice. The most distinctive adverse event associated with sorafenib treatment was systemic hypertension, which developed in approximately half of the treated dogs. This finding is consistent with the known on-target toxicity of VEGFR inhibition observed in human patients receiving sorafenib and other anti-angiogenic agents^39^. Hypertension was effectively controlled using antihypertensive agents, such as calcium channel blockers or angiotensin receptor blockers, even when the systolic blood pressure exceeded 170 mmHg, and allowed the continuation of sorafenib treatment. One dog (Case S4) developed grade 3 neurological signs and was suspected of having hypertensive encephalopathy, exhibited severe hypertension (systolic blood pressure 240 mmHg), dizziness, ataxia, and horizontal nystagmus; however, magnetic resonance imaging (MRI) revealed no structural abnormalities. After receiving amlodipine treatment (0.24 mg/kg once daily), the dog’s systolic blood pressure improved to 150–160 mmHg within 1 month, and all neurological signs resolved, which supports the diagnosis of hypertensive encephalopathy. This case highlights the importance of careful monitoring of blood pressure and timely intervention during sorafenib treatment. Interestingly, this dog achieved a partial response and survived for 501 days despite developing severe hypertension. Although conclusions cannot be drawn from a single case, this observation illustrates the potential coexistence of pronounced on-target toxicity and sustained clinical benefit during sorafenib treatment.

This study found that sorafenib-induced systemic hypertension was associated with prolonged OS in dogs with urothelial carcinoma, which is consistent with observations in clinical studies of human patients^31,32^. Inhibiting VEGFR signaling in vascular endothelial cells reduces nitric oxide and prostacyclin production while increasing vasoconstrictor endothelin-1^39^. These effects lead to increased systemic vascular resistance and hypertension. Multiple clinical studies in human oncology have demonstrated improved outcomes in patients who develop hypertension during treatment with VEGFR-targeting agents, such as sorafenib, sunitinib, and bevacizumab^27,28,39^, which supports the role of treatment-induced hypertension as a surrogate indicator of adequate drug exposure and sustained inhibition of tumor angiogenesis. To our knowledge, this is the first prospective veterinary clinical trial to demonstrate that on-target toxicity of molecular-targeted therapy is associated with prolonged survival. This finding highlights the value of naturally occurring canine cancer models for verifying the relationship between adverse events and therapeutic effects, which are difficult to evaluate in rodent experimental models.

Although renal adverse events were anticipated owing to the high incidence of treatment-induced hypertension, the incidence and severity of renal dysfunction were lower than expected. Only one dog (Case S42) experienced grade 3 acute kidney injury; however, renal function improved with fluid therapy and piroxicam withdrawal, whereas the sorafenib treatment was continued. This suggests that the renal toxicity was likely caused by piroxicam rather than by sorafenib. This finding is consistent with previous observations that nonsteroidal anti-inflammatory drugs significantly contribute to adverse renal events in dogs with urothelial carcinoma. Compared to lapatinib-based therapy^26^, sorafenib treatment is associated with a lower incidence of gastrointestinal adverse events, such as vomiting, diarrhea, and anorexia, as well as fewer elevations in liver enzymes. Dermatological adverse events, including hyperpigmentation, pruritus, and alopecia, were occasionally observed in this study, similar to those reported with lapatinib treatment^26^, but were generally mild and did not require intervention. Collectively, these safety data suggest that sorafenib/piroxicam treatment is well tolerated in elderly dogs and exhibits lower hepatotoxicity and nephrotoxicity than conventional cytotoxic chemotherapy. This superior tolerability is advantageous for both canine and human patients with comorbidities (particularly chronic kidney disease), which supports the role of sorafenib as a practical and accessible targeted therapy.

Besides its hemodynamic effects, VEGFR inhibition may modulate the tumor microenvironment and enhance antitumor efficacy. Beyond vascular normalization and attenuation of hypoxia-driven tumor progression, accumulating evidence indicates that VEGFR signaling directly contributes to tumor-associated immune suppression^40^. Previous studies using canine urothelial carcinoma cell lines and mouse xenograft models demonstrated that VEGFR stimulation enhances CX3CL1 expression in tumor cells and that MDSCs express the CX3CL1 receptor CX3CR1. This axis enables chemotactic recruitment of MDSCs into the tumor microenvironment. Sorafenib disrupted the VEGFR–CX3CL1 axis, suppressed MDSC migration, and exerted antitumor effects both in vitro and in vivo^22,23^. Consistent with these findings, the present clinical trial revealed that higher tumor/urinary CX3CL1 levels and a greater reduction in circulating PMN-MDSCs following sorafenib treatment were associated with prolonged PFS and OS in univariate analyses. Notably, high tumor *CX3CL1* mRNA expression was independently associated with improved OS in multivariable analyses, suggesting that tumor-derived CX3CL1 may represent a robust prognostic biomarker reflecting sorafenib-mediated modulation of the tumor immune microenvironment. The reduction rate of circulating PMN-MDSCs was not included in the multivariable analysis due to the limited sample size; therefore, its independent prognostic value requires further validation in larger cohorts. Taken together, our results suggest that sorafenib inhibits VEGFR signaling and exerts multifaceted antitumor effects by suppressing tumor angiogenesis and modulating chemokine-mediated immune responses in canine urothelial carcinoma.

The BRAF^V595E^ mutation plays a role in tumor cell proliferation and the development of an immunosuppressive tumor microenvironment in canine urothelial carcinoma. Previous RNA sequencing analyses have suggested that canine urothelial carcinomas with the BRAF^V595E^ mutation exhibit transcriptional patterns associated with immune suppression^18^. Furthermore, the BRAF^V595E^ mutation increases the production of prostaglandin E2 (PGE2)^41^—a lipid mediator that promotes differentiation and expansion of MDSCs and regulatory T cells (Tregs). The BRAF^V595E^ mutation enhances the expression of CCL17—a chemokine that facilitates the recruitment of CCR4^+^ Tregs to the tumor microenvironment—and leads to immune evasion and tumor progression^37,42^. A previous clinical trial of the BRAF inhibitor vemurafenib in dogs with BRAF^V595E^ mutation-positive urothelial carcinoma suggested that BRAF signaling plays an immunomodulatory role^36^. The trial found that BRAF inhibition increased intratumoral CD8^+^ T-cell infiltration, resulting in an “immune-hot” tumor phenotype within 1 month of treatment initiation. This immunological shift correlates with therapeutic efficacy^36^. Although the present study showed that the BRAF^V595E^ mutation was associated with OS in univariate analyses, this association was not retained after adjustment for covariates in multivariable models. This finding suggests that the prognostic impact of BRAF^V595E^ mutation may be influenced by other tumor- or treatment-related factors rather than acting as an independent determinant of outcome under sorafenib therapy. Nevertheless, given its known role in shaping the tumor immune microenvironment, further investigation is warranted to clarify whether BRAF signaling contributes to therapeutic responsiveness through immunomodulatory mechanisms. Previously, we demonstrated that targeting Tregs with mogamulizumab, a monoclonal antibody against CCR4, induced clinically meaningful antitumor responses in dogs with urothelial carcinoma^37,43^. These observations suggest that the combination of sorafenib and mogamulizumab could synergistically enhance antitumor immunity by suppressing BRAF-driven CCL17 production and depleting CCR4-positive Tregs. Further investigation of this combination strategy as a rational immunomodulatory approach for malignancies with BRAF mutations is warranted.

One possible explanation for the observed efficacy of sorafenib/piroxicam therapy in some BRAF^V595E^-negative cases is the presence of alternative molecular abnormalities, such as HER2 overexpression or *ERBB2* amplification. In previous studies, we demonstrated that approximately 60% of canine urothelial carcinomas overexpress HER2 and that approximately 30% harbor *ERBB2* gene amplification^44,45^. We also reported that treatment with the HER2 inhibitor lapatinib provides clinical benefit in dogs with urothelial carcinoma^26^. Since HER2 signaling converges on the RAF/MEK/ERK pathway, inhibition of downstream RAF signaling by sorafenib may contribute to antitumor activity in tumors driven by HER2 activation. However, HER2 expression and *ERBB2* copy number alteration were not evaluated in the present study. Therefore, the potential contribution of HER2 signaling to treatment response remains unclear and warrants further investigation in future studies incorporating HER2 expression and *ERBB2* genomic analyses.

This study has some limitations. First, this was a prospective single-arm study that evaluated the combination of sorafenib and piroxicam without a concurrent control group or randomization. Therefore, direct comparisons with other treatment modalities or definitive attribution of clinical benefits to sorafenib cannot be made, and the potential influence of confounding factors cannot be fully excluded. Although no significant differences in baseline clinical characteristics were identified between the sorafenib/piroxicam cohort and the historical control cohort (Supplementary Table S2), residual confounding cannot be completely excluded. In addition, because the control cohort was historical rather than concurrent, temporal bias related to changes in supportive care, ancillary treatments, or owner compliance cannot be excluded. Randomized controlled trials or well-designed comparative studies are required to conclusively establish the efficacy of sorafenib in dogs with urothelial carcinoma. Second, although this study examined sorafenib-induced adverse events, clinical outcomes, and immune-related biomarkers, comprehensive longitudinal analyses of intratumoral immune cell populations were not performed. Direct evaluation of the changes in tumor-infiltrating MDSCs, Tregs, and effector T cells before and after treatment would further strengthen the proposed immunomodulatory mechanisms of sorafenib. In addition, longitudinal analyses of circulating MDSCs were only available in 18 of the 43 dogs enrolled in the present study because paired pre- and post-treatment blood samples were required, and the flow cytometric evaluation of MDSCs necessitated the collection of relatively large blood volumes at both time points, which was not feasible in all dogs. Therefore, the findings regarding PMN-MDSC dynamics should be considered exploratory and require validation in larger independent cohorts. Third, although this study evaluated several key molecular targets of sorafenib, including BRAF mutation status and components of the VEGF and PDGF signaling pathways, it did not fully evaluate the range of sorafenib target molecules, such as CRAF, FLT3, KIT, and RET. Previous immunohistochemical studies of canine urothelial carcinoma have shown that VEGFR2 and PDGFRβ are widely expressed in tumor tissues in most cases^20,21^, which supports the biological relevance of these pathways in this disease. In contrast, KIT expression is infrequent and, when present, is expressed at low levels in canine urothelial carcinoma^20,21^. Furthermore, no published study has demonstrated an association between canine urothelial carcinoma and other known sorafenib targets, such as CRAF, RET, or FLT3. Given the multi-kinase inhibitory profile of sorafenib, a comprehensive evaluation of these and other signaling pathways may help further elucidate the molecular determinants of the therapeutic response and refine biomarker-driven treatment strategies in future clinical trials. In addition, no suitable human cohort receiving VEGFR-targeted therapies was available for external validation of the biomarkers identified in this study. Future comparative oncology studies integrating canine and human bladder cancer datasets will be important to determine whether biomarkers identified in sorafenib-treated dogs, including CX3CL1, have conserved biological and clinical relevance across species. In particular, validation in human cohorts receiving VEGFR-targeted therapies may help clarify the translational significance of these findings. Fourth, biomarker stratification analyses were performed using median values as prespecified cutoffs to classify cases into high- and low-expression groups. Although this approach avoids data-driven threshold optimization and reduces the risk of overfitting in relatively small cohorts, the biological relevance of these cutoffs remains uncertain. In addition, no independent external validation cohort was available to confirm the prognostic significance of the evaluated biomarkers. Therefore, the prognostic value and optimal thresholds of these biomarkers should be considered exploratory and require validation in larger independent prospective cohorts.

In conclusion, sorafenib/piroxicam combination therapy is a promising therapeutic option for dogs with urothelial carcinoma, which demonstrated clinically significant antitumor activity and an acceptable safety profile. This prospective clinical trial provides the first evidence in veterinary medicine that an on-target toxicity of molecular-targeted therapy, systemic hypertension, is associated with prolonged survival, supporting the biological relevance of effective VEGFR pathway inhibition. In addition, tumor *CX3CL1* expression emerged as a potential treatment-related biomarker associated with improved survival, suggesting that sorafenib-mediated modulation of the VEGFR–CX3CL1–MDSC axis may contribute to therapeutic efficacy. Given the shared molecular characteristics between canine and human malignancies, this naturally occurring canine model provides valuable insights for comparative oncology and supports the translational development of biomarker-guided sorafenib-based strategies for muscle-invasive bladder cancer.

## Methods

### Ethical statements

The Animal Care and Clinical Research Committee of VMC-UT approved this study as a prospective, open-label, single-arm, single-center canine clinical trial (approval no. VMC2019-03). Written informed consent was obtained from all dog owners. All experimental methods were performed in accordance with the ARRIVE guidelines. All methods were performed in accordance with relevant guidelines and regulations.

### Canine clinical trial design and interventions

A Phase II clinical trial in dogs was performed at the VMC-UT. Client-owned dogs with histologically confirmed muscle-invasive urothelial carcinoma were enrolled, and those that had previously undergone surgery, chemotherapy, or radiotherapy were excluded. Prior to enrollment, all dogs underwent a physical examination, blood pressure measurement, laboratory testing (complete blood count, serum biochemical analysis, and urinalysis), 3-view thoracic radiography, 2-view abdominal radiography, and abdominal ultrasonography within 7 days of study entry. Dogs with significant comorbidities were excluded. Other exclusion criteria were complete urethral obstruction or hydronephrosis owing to tumor invasion, serum creatinine > 3.0 mg/dL, total bilirubin > 2.0 mg/dL, hematocrit < 25%, or platelet count < 50,000/µL. Before treatment, tumor staging was determined according to the WHO criteria for canine bladder cancer^24,25^. In this trial, sorafenib (Nexavar^®^, 5–10 mg/kg; Bayer, Leverkusen, Germany) and piroxicam (Baxo^®^, 0.3 mg/kg; FUJIFILM Toyama Chemical, Tokyo, Japan) were administered orally to dogs with urothelial carcinoma once every 24 h. Piroxicam is a standard drug used to treat canine urothelial carcinoma^24^. The dose and dosing interval for sorafenib were determined based on repeated-dose toxicity studies in dogs, as described in the Nexavar^®^ interview form. Treatment was continued until disease progression, the development of unacceptable toxicity, or discontinuation by the owner. If the owner wished to continue treatment for perceived clinical benefit and to assess possible non-conventional responses, the dogs were permitted to remain on sorafenib after progression. This study did not include placebo controls, blinding, or randomization.

### Clinical assessment

The clinical response and treatment-related toxicity were evaluated in all dogs at intervals of at least 4 weeks. The assessments included owner-reported observations, physical examinations, blood pressure measurements, complete blood counts, serum biochemical analyses, 3-view thoracic radiography, and abdominal ultrasonography. All ultrasonographic examinations and tumor measurements were performed by the same investigator (SM) according to a standardized protocol^46,47^, and the tumor volume was estimated and recorded. Ultrasonography was selected as the imaging modality because it does not require general anesthesia (as is required for computed tomography in dogs). To minimize variability associated with bladder filling, all ultrasonographic examinations at the initial visit were performed after transurethral catheterization and instillation of 25 mL sterile saline into the urinary bladder. At follow-up examinations, owners were instructed not to allow urination immediately before hospital visits, and ultrasonographic evaluations were generally performed at least 2 h after the last urination. In cases where dogs urinated during the waiting period before examination and the urinary bladder was nearly empty at the time of imaging, sterile saline was instilled via a urinary catheter before ultrasonographic assessment. Tumor responses were categorized as follows: CR, disappearance of all measurable lesions; PR, ≥ 50% reduction in tumor volume without new lesions; SD, < 50% change in tumor volume without new lesions; and PD, ≥ 50% increase in tumor volume or the appearance of new lesions^26,37,48,49^. PFS was defined as the interval from treatment initiation to PD or death as assessed at the study endpoint (May 31, 2025). OS was defined as the interval from treatment initiation to the death of the dog at the study endpoint. Adverse events were evaluated according to the Veterinary Cooperative Oncology Group (VCOG) criteria^50^.

Blood pressure was measured using an oscillometric technique (BSM-3592 Life Scope VS, Nihon Kohden, Tokyo, Japan). Six measurements were recorded at each assessment and the initial SBP readings were discarded. The SBP recorded as a representative of that event was the mean of the last five values obtained. Hypertension was defined as an SBP of at least 160 mmHg, as previously recommended by the ACVIM consensus statements^51^. Hypertension following sorafenib administration was defined as an increase in SBP above baseline levels, with a posttreatment SBP > 160 mmHg. Antihypertensive therapy was initiated when blood pressure was ≥ 170 mmHg, a level consistent with moderate hypertension associated with target organ damage^51^.

### Biomarkers

For biomarker assessment, fresh tumor and urine samples were collected before treatment for the quantification of VEGF-A, PDGF-BB, CX3CL1, and BRAF^V595E^ mutation. Urinary levels of canine VEGF-A, PDGF-BB, and CX3CL1 were determined in urinary supernatants using ELISA kits from Abcam (Cambridge, UK), RayBiotech (Peachtree Corners, GA, USA), and BlueGene Biotech (Shanghai, China), respectively. Urinary creatinine concentration was measured using the LabAssay Creatinine kit (Fujifilm Wako Pure Chemical, Osaka, Japan). To account for variations in urine concentration, the levels of VEGF-A, PDGF-BB, and CX3CL1 were normalized and expressed as pg/mg of creatinine^22^. BRAF^V595E^ mutation was examined by digital PCR using genomic DNA isolated from urine sediments, as previously described^42^. Tumor expression levels of *VEGFA*, *PDGFB*, and *CX3CL1* mRNA were quantified using two-step real-time PCR (Applied Biosystems, Waltham, MA, USA)^22^. *ACTB* and *GAPDH* were used as reference genes. The primer pair sequences are shown in Supplementary Table S6. Archived snap-frozen normal bladder tissues obtained from 7 healthy beagles euthanized for other experimental purposes were used as a normal control. This control group included 2 females (all intact) and 5 males (all intact), aged 14–30 months (median, 29 months). These dogs exhibited no clinical signs. Routine urinalysis and blood examinations, including a complete blood count and measurements of blood urea nitrogen, creatinine, alanine aminotransferase, and alkaline phosphatase levels, showed no abnormalities. Histopathological examination confirmed no abnormalities in the bladder.

Circulating MDSCs before and after sorafenib administration were evaluated by flow cytometry using peripheral blood mononuclear cells isolated from EDTA blood samples. In accordance with previous studies^29^, CD11b⁺ CD14⁻ MHCII⁻ cells were defined as PMN-MDSCs, and CD11b⁺ CD14⁺ MHCII⁻ cells were defined as M-MDSCs for analysis. Flow cytometry was performed as previously described^29^.

### Endpoints and statistical analyses

The primary endpoint of this study was PFS. The secondary endpoints were OS, response rates, adverse events, and outcomes of subsets depending on sorafenib dose, sorafenib-induced hypertension, and biomarker status (tumor expression levels of *VEGFA*, *PDGFB*, and *CX3CL1*, urinary levels of VEGF-A, PDGF-BB, and CX3CL1, BRAF^V595E^ mutation, PMN-MDSCs, and M-MDSCs). A power analysis was performed to estimate the number of dogs needed to detect a statistically significant difference in PFS between the sorafenib/piroxicam group and historical control (piroxicam alone) group using Power and Sample Size Calculation software v.3.1.6 (https://biostat.app.vumc.org/wiki/Main/PowerSampleSize)^52^. Based on a previous study^26^, the duration of PFS in dogs treated with piroxicam alone was 90 days. A minimum of 38 dogs in each group were required for enrollment based on an alpha level of 0.05 to detect a 2-fold longer median PFS in the sorafenib/piroxicam group with 80% power. The Mann–Whitney *U* test for continuous variables and Fisher’s exact test for categorical variables. The Cochran–Armitage test for trend was used to evaluate the association between clinical response and sorafenib dose, hypertension after treatment, or biomarker status. Cases were divided into high- and low-dose groups based on the median sorafenib dose, and associations with adverse events and treatment-associated hypertension were assessed using Fisher’s exact test. Survival curves were generated using the Kaplan–Meier method. The PFS and OS in dogs treated with sorafenib and piroxicam in this clinical trial were compared with those in 42 dogs in the historical control group treated with piroxicam alone in our previous study^26^ using the log-rank test. To identify prognostic factors associated with survival under sorafenib treatment, univariate Cox proportional hazards regression analyses were performed for the following variables: age, sex, BRAF^V595E^ mutation status, posttreatment hypertension, tumor *VEGFA* mRNA expression, tumor *PDGFB* mRNA expression, tumor *CX3CL1* mRNA expression, urinary VEGF-A concentration, urinary PDGF-BB concentration, urinary CX3CL1 concentration, baseline PMN-MDSC proportion, baseline M-MDSC proportion, PMN-MDSC reduction rate, and M-MDSC reduction rate. Variables showing statistical significance in the univariate Cox proportional hazards model were considered for inclusion in the multivariate models. JMP Pro v. 11.2 (SAS Institute, Cary, NC, USA) was used for statistical analyses. Statistical significance was set at *P* < 0.05.

## Supplementary Information

Below is the link to the electronic supplementary material.


Supplementary Material 1



Supplementary Material 2


## Data Availability

All data supporting this study’s findings are available within the paper and its supplementary information files.
