# Supplementary material for: Sorafenib/piroxicam therapy with biomarker-defined survival benefit in canine muscle-invasive urothelial carcinoma
Source: Sci Rep. 2026 Sep 1;16:24028. doi: 10.1038/s41598-026-64955-1 (PMC13534594; doi:10.1038/s41598-026-64955-1)
Supplement: Supplementary file 1 [file 41598_2026_64955_MOESM1_ESM.pdf]

Supplementary Materials for

Sorafenib/piroxicam therapy with biomarker-defined survival benefit in canine muscle-invasive urothelial carcinoma

Shingo Maeda*, Shohei Yokota, Mao Komori, Yuko Goto-Koshino, James K. Chambers, Kazuyuki Uchida, Tomohiro Yonezawa, Yasuyuki Momoi

*Corresponding author. E-mail: amaeda@g.ecc.u-tokyo.ac.jp

The PDF file includes:

Table S1. Characteristics of dogs with urothelial carcinoma in the clinical trial.

Table S2. Comparison of baseline clinical characteristics between dogs treated with sorafenib/piroxicam and the historical piroxicam alone cohort.

Table S3. Association of sorafenib dose with treatment-related adverse events in dogs treated with sorafenib and piroxicam.

Table S4. Association of sorafenib dose with posttreatment hypertension in dogs treated with sorafenib and piroxicam.

Table S5. Univariate analysis for survival with Cox proportional hazards model.

Table S6. Primer pair sequences used for RT-qPCR.

Fig. S1. Association of sorafenib dose with the clinical responses and survival in dogs treated with sorafenib and piroxicam.

Fig. S2. Association of VEGF expression levels with the clinical responses and survival in dogs treated with sorafenib and piroxicam.

Fig. S3. Association of PDGF expression levels with the clinical responses and survival in dogs treated with sorafenib and piroxicam.

Fig. S4. Association of myeloid-derived suppressor cells (MDSCs) with the clinical responses and survival in dogs treated with sorafenib and piroxicam.

Table S1. Characteristics of dogs with urothelial carcinoma in the clinical trial.

Case ID	Age (years)	Sex[a]	Breed	TNM classification	$BRAF^{V595E}$ mutation	$BRAF^{V595E}$ mutation VAF (%)	Treatment	Sorafenib dose (mg/kg)	Baseline SBP[b] (mmHg)	Post treatment SBP (mmHg)	Post treatment hypertension	Response[c]	PFS[d] (days)	OS[e] (days)
S1	10.8	FN	Border Collie	T2N0M0	Mutation	32.0	Sorafenib/piroxicam	4.8	142	159	–	PR	120	177
S2	15.6	FN	Boston Terrier	T2N1M1	Mutation	44.8	Sorafenib/piroxicam	5.6	146	166	+	PR	140	194
S3	9.9	MN	Miniature Dachshund	T2N1M0	Wild-type	0	Sorafenib/piroxicam	8.7	140	210	+	PR	161	284
S4	12.1	FN	Shetland Sheepdog	T2N1M0	Mutation	44.3	Sorafenib/piroxicam	4.7	176	240	+	PR	119	501
S5	17.1	MN	Pomeranian	T2N1M0	Mutation	29.0	Sorafenib/piroxicam	7.7	142	145	–	PR	248	248
S6	11.6	FN	Border Collie	T3N1M0	Wild-type	0	Sorafenib/piroxicam	4.9	173	169	–	PD	84	103
S7	13.6	FN	Miniature Dachshund	T2N0M0	Wild-type	0	Sorafenib/piroxicam	7.7	150	183	+	SD	327	698
S8	10.6	MN	Miniature Dachshund	T2N0M0	Mutation	35.2	Sorafenib/piroxicam	8.3	140	179	+	PR	548	1168
S9	11.3	MN	Papillon	T2N0M0	Mutation	48.1	Sorafenib/piroxicam	6.7	140	169	+	PR	291	943
S10	14.8	FN	Miniature Dachshund	T2N0M0	Mutation	56.2	Sorafenib/piroxicam	7.4	133	135	–	PR	301	399
S11	15.5	FN	Miniature Dachshund	T3N0M0	Mutation	40.5	Sorafenib/piroxicam	7.3	144	156	–	PR	175	788
S12	9.8	MN	Shih Tzu	T2N0M0	Mutation	8.6	Sorafenib/piroxicam	5.1	136	143	–	PD	31	214
S13	10.9	FN	Toy Poodle	T2N1M0	Mutation	48.5	Sorafenib/piroxicam	9.5	161	159	–	SD	210	798
S14	11.6	FN	Shetland Sheepdog	T2N1M0	Mutation	25.9	Sorafenib/piroxicam	6.3	120	132	–	PR	112	145
S15	12.2	MN	West Highland White Terrier	T2N1M0	Mutation	41.6	Sorafenib/piroxicam	4.9	137	181	+	SD	139	160
S16	7.6	MI	Miniature Dachshund	T2N0M0	Mutation	38.3	Sorafenib/piroxicam	6.4	151	188	+	SD	665	1723
S17	14.4	MN	Toy Poodle	T2N0M1	Mutation	7.2	Sorafenib/piroxicam	7.4	147	168	+	PR	94	277
S18	15.3	FN	Miniature Dachshund	T2N0M0	Wild-type	0	Sorafenib/piroxicam	8.1	152	148	–	PR	266	407
S19	9.3	FN	Border Collie	T2N0M0	Wild-type	0	Sorafenib/piroxicam	6.2	146	156	–	PR	306	535
S20	9.7	FN	Pomeranian	T2N0M0	Mutation	44.1	Sorafenib/piroxicam	8.5	150	193	+	SD	252	515
S21	8.4	MN	Toy Poodle	T2N0M0	Wild-type	0	Sorafenib/piroxicam	9.1	135	142	–	PR	147	555
S22	7.9	FN	Norfolk Terrier	T2N0M0	Mutation	31.5	Sorafenib/piroxicam	7.2	136	164	+	PR	379	536
S23	14.0	FN	Scottish Terrier	T2N0M1	Mutation	33.1	Sorafenib/piroxicam	5.1	125	149	–	SD	166	327
S24	13.5	FN	Border Collie	T2N0M1	Wild-type	0	Sorafenib/piroxicam	6.1	160	144	–	PR	135	135
S25	12.1	MN	Papillon	T2N0M1	Mutation	14.1	Sorafenib/piroxicam	10	150	181	+	PR	220	563
S26	13.7	FN	Mongrel	T2N0M0	Wild-type	0	Sorafenib/piroxicam	9	140	177	+	SD	142	404
S27	14.0	FN	Maltese	T2N0M0	Wild-type	0	Sorafenib/piroxicam	8.1	139	176	+	PR	435	834
S28	12.2	MN	Toy Poodle	T2N0M1	Mutation	3.1	Sorafenib/piroxicam	4.6	138	145	–	PD	84	164
S29	11.0	FN	Pembroke Welsh Corgi	T3N0M1	Mutation	46.6	Sorafenib/piroxicam	6.7	126	126	–	SD	119	338
S30	11.3	MN	Shetland Sheepdog	T2N0M1	Mutation	40.5	Sorafenib/piroxicam	9.2	157	195	+	PR	434	689
S31	14.1	FN	Pembroke Welsh Corgi	T2N0M0	Wild-type	0	Sorafenib/piroxicam	5.4	120	131	–	SD	151	151
S32	16.1	FI	Miniature Dachshund	T2N0M0	Wild-type	0	Sorafenib/piroxicam	10.1	174	175	–	PR	174	230
S33	8.8	FN	Mongrel	T3N0M0	Mutation	43.3	Sorafenib/piroxicam	7.5	153	148	–	PR	546	722
S34	13.7	FN	Mongrel	T3N1M0	Wild-type	0	Sorafenib/piroxicam	5.3	128	150	–	PD	56	144
S35	13.7	FN	Miniature Dachshund	T3N1M0	Wild-type	0	Sorafenib/piroxicam	9.9	149	153	–	PR	355	502
S36	15.6	FN	Maltese	T3N0M0	Mutation	42.9	Sorafenib/piroxicam	8.8	135	162	+	SD	400	1334
S37	12.3	FN	Bernese Mountain Dog	T3N0M0	Wild-type	0	Sorafenib/piroxicam	6.8	134	135	–	PR	252	297
S38	8.4	FN	Mongrel	T2N0M0	Wild-type	0	Sorafenib/piroxicam	7.9	148	175	+	PR	173	592
S39	11.0	FN	Mongrel	T2N0M0	Mutation	18.8	Sorafenib/piroxicam	7.2	127	159	–	PR	255	1313
S40	9.1	MN	Shetland Sheepdog	T2N0M0	Mutation	33.9	Sorafenib/piroxicam	8.7	111	164	+	PR	119	1296
S41	10.2	MN	Cavalier King Charles Spaniel	T2N0M0	Mutation	31.7	Sorafenib/piroxicam	7.2	133	179	+	SD	385	1292
S42	13.3	MN	Shetland Sheepdog	T2N0M0	Mutation	29.5	Sorafenib/piroxicam	6.9	145	180	+	PR	246	350
S43	10.9	FN	Shetland Sheepdog	T2N0M1	Wild-type	0	Sorafenib/piroxicam	8.7	135	169	+	PD	91	158

[a]FN, female neutered; FI, female intact; MN, male neutered; MI, male intact. [b]SBP, systolic blood pressure. [c]CR, complete remission; PR, partial response; SD, stable disease; PD, progressive disease. [d]PFS, progression-free survival. [e]OS, overall survival.

Table S2. Comparison of baseline clinical characteristics between dogs treated with sorafenib/piroxicam and the historical piroxicam alone cohort.

	Sorafenib/piroxicam (*n* = 43)	Piroxicam (*n* = 42)	
**Characteristics**	**No. of dogs (%)**	**No. of dogs (%)**	***P* value**
**Age[a] (years)**			
Median	12.1	11.9	0.58
Range	7.6–17.1	7–15.9	
**Body weight[a] (kg)**			
Median	6.9	6.5	0.91
Range	2.7–29.5	1.3–37.6	
**Sex[b]**			
Female neutered	27 (62.8)	21 (50.0)	1.00
Female intact	1 (2.3)	6 (14.3)	
Male neutered	14 (32.6)	10 (23.8)	
Male intact	1 (2.3)	5 (11.9)	
**Muscle-invasive tumor[b] (T2 or T3)**	43 (100)	42 (100)	1.00
**T-stage classification[b]**			
T2	35 (81.4)	38 (90.5)	0.35
T3	8 (18.6)	4 (9.5)	
**Tumor location[b]**			
Vesical apex	5 (11.6)	6 (14.3)	0.63
Vesical body	11 (25.6)	12 (28.6)	
Vesical trigone	25 (58.1)	22 (52.4)	
Urethra involvement	21 (48.8)	12 (28.6)	
Prostate involvement	5 (11.6)	2 (4.8)	
**Tumor volume[a] (mm^3)**			
Median	2,834	2,835	0.91
Range	163–25,833	217–29,752	
**Metastasis[b]**			
Any	18 (41.9)	13 (31.0)	0.37
Lymph node	10 (23.3)	11 (26.2)	
Lung	9 (20.9)	3 (7.1)	
**BRAF gene mutation[b]**			
Wild-type	16 (37.2)	11 (26.2)	0.35
$BRAF^{V595E}$ mutation	27 (62.8)	31 (73.8)	

[a]The Mann–Whitney *U* test.

[b]Fisher's exact test.

Table S3. Association of sorafenib dose with treatment-related adverse events in dogs treated with sorafenib and piroxicam.

	**Any adverse events (*n* = 43)**		
**Sorafenib dose group**	**Yes**	**No**	***P* value[a]**
**High-dose (*n* = 21)**	18	3	1.000
**Low-dose (*n* = 22)**	18	4	
**Total**	36	7	

[a]Fisher's exact test.

Table S4. Association of sorafenib dose with posttreatment hypertension in dogs treated with sorafenib and piroxicam.

	Posttreatment hypertension (*n* = 43)		
**Sorafenib dose group**	**Yes**	**No**	***P* value[a]**
**High-dose (*n* = 21)**	13	8	0.131
**Low-dose (*n* = 22)**	8	14	
**Total**	21	22	

[a]Fisher's exact test.

Table S5. Univariate analysis for survival with Cox proportional hazards model

Characteristics	Progression-free survival		Overall survival	
	HR (95% CI)	*P* value	HR (95% CI)	*P* value
**Age**				
<12 years	Reference		Reference	
≥12 years	1.48 (0.78–2.79)	0.225	2.12 (1.09–4.15)	**0.028**
**Sex**				
Male	Reference		Reference	
Female	1.32 (0.68–2.57)	0.415	1.52 (0.76–3.08)	0.235
**BRAF gene mutation**				
Wild-type	Reference		Reference	
$BRAF^{V595E}$ mutation	0.72 (0.38–1.36)	0.309	0.50 (0.25–0.99)	**0.049**
**Posttreatment hypertension**				
No	Reference		Reference	
Yes	0.55 (0.29–1.04)	0.066	0.43 (0.22–0.84)	**0.013**
**Tumor *VEGFA* mRNA**				
Low	Reference		Reference	
High	1.12 (0.58–2.19)	0.734	1.05 (0.51–2.17)	0.888
**Tumor *PDGFB* mRNA**				
Low	Reference		Reference	
High	0.92 (0.47–1.79)	0.811	0.83 (0.41–1.71)	0.619
**Tumor *CX3CL1* mRNA**				
Low	Reference		Reference	
High	0.45 (0.22–0.88)	**0.021**	0.31 (0.15–0.65)	**0.002**
**Urinary VEGF-A protein**				
Low	Reference		Reference	
High	1.01 (0.48–2.14)	0.981	1.09 (0.49–2.43)	0.840
**Urinary PDGF-BB protein**				
Low	Reference		Reference	
High	1.45 (0.69–3.03)	0.323	1.40 (0.62–3.14)	0.414
**Urinary CX3CL1 protein**				
Low	Reference		Reference	
High	0.45 (0.21–0.94)	**0.033**	0.24 (0.10–0.55)	**0.001**
**Baseline PMN-MDSCs**				
Low	Reference		Reference	
High	0.74 (0.28–1.92)	0.530	1.05 (0.40–2.75)	0.923
**Baseline M-MDSCs**				
Low	Reference		Reference	
High	1.90 (0.66–5.49)	0.235	1.29 (0.48–3.44)	0.617
**PMN-MDSC reduction rate**				
Low	Reference		Reference	
High	0.31 (0.11–0.90)	**0.032**	0.12 (0.02–0.58)	**0.009**
**M-MDSC reduction rate**				
Low	Reference		Reference	
High	2.25 (0.73–6.88)	0.155	0.87 (0.33–2.26)	0.770

HR, hazard ratio; CI, confidence Interval.

Table S6. Primer pair sequences used for RT-qPCR.

Primer set	GenBank accession number		Primer sequence (5′–3′)
CX3CL1	AB648939	Forward	CTT CCT TGG CCT CCT CTT CT
		Reverse	GGC ACC AGG ACA TAC GAG TT
VEGFA	NM_001003175	Forward	GCA ACT CCG GCA GAA GCA T
		Reverse	TGG CGA TCC AAT TCC AAG AG
PDGFB	NM_001003383	Forward	GGC CGA GTT GGA CCT GAA T
		Reverse	TCT ATG AGG CGC CGT GAG AT
ACTB	AF021873	Forward	GCG CAA GTA CTC TGT GTG GAT
		Reverse	GTC GTA CTC CTG CTT GCT GAT
GAPDH	AB038240	Forward	CAT GTT TGT GAT GGG CGT GAA CCA
		Reverse	TTT GGC TAG AGG AGC CAA GCA GTT

VEGFA, vascular endothelial growth factor A; *PDGFB*, platelet derived growth factor B; *ACTB*, actin beta; *GAPDH*, glyceraldehyde-3-phosphate dehydrogenase.

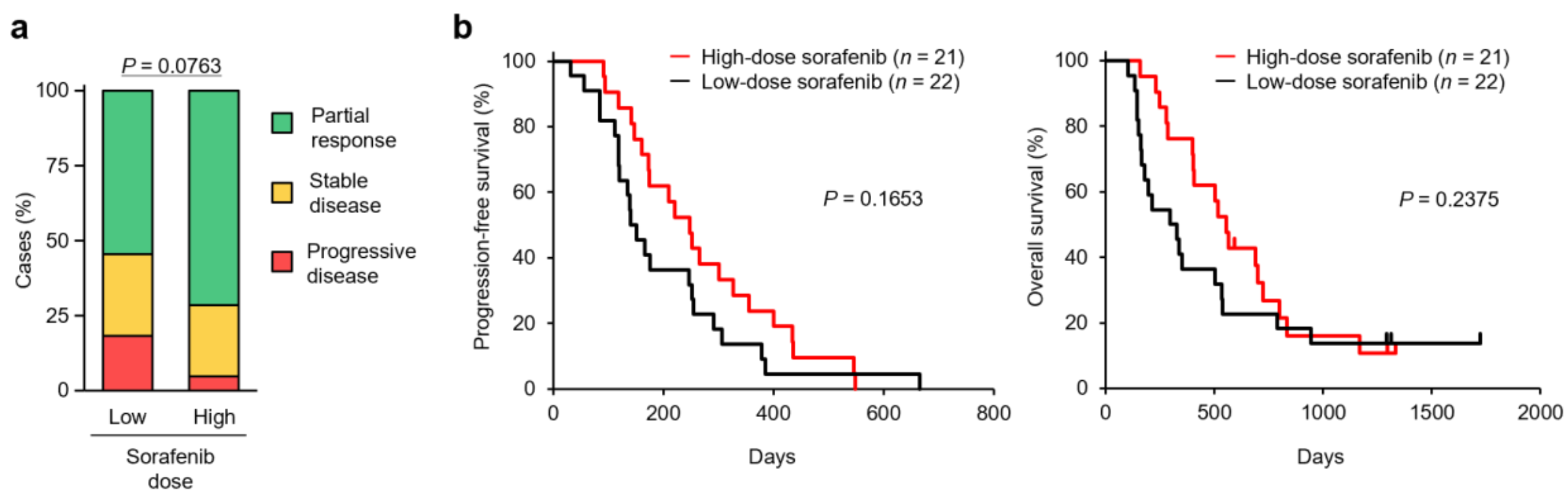

Figure S1. Association of sorafenib dose with the clinical responses and survival in dogs treated with sorafenib and piroxicam. (**a**) Association between sorafenib dose and clinical response (*n* = 43). Cases were classified as either high-dose (*n* = 21) or low-dose (*n* = 22) groups based on the median sorafenib dose, Cochran–Armitage test. (**b**) Kaplan–Meier curves of progression-free survival (left) and overall survival (right) according to sorafenib dose in dogs treated with sorafenib and piroxicam (*n* = 43). Cases were classified as either high-dose (*n* = 21) or low-dose (*n* = 22) groups based on the median sorafenib dose, log-rank test.

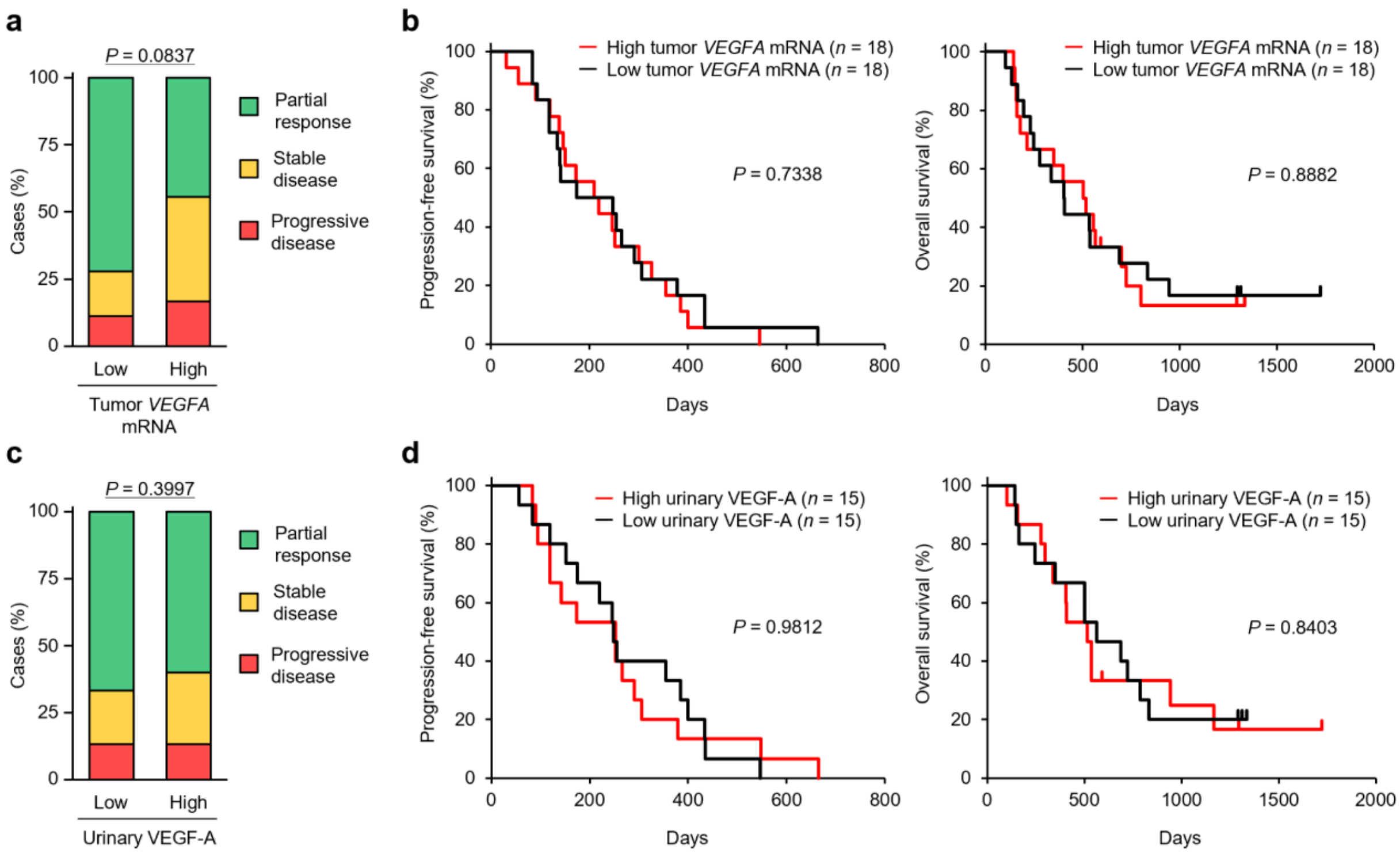

Figure S2. Association of VEGF expression levels with the clinical responses and survival in dogs treated with sorafenib and piroxicam. (**a**) Association of baseline tumor *VEGFA* mRNA expression with clinical response (n = 36). Cases were classified as either high or low tumor *VEGFA* mRNA (n = 18 each) according to the median, Cochran–Armitage test. (**b**) Kaplan–Meier curves of progression-free survival (left) and overall survival (right) according to baseline tumor *VEGFA* mRNA expression in dogs treated with sorafenib and piroxicam (n = 36). Cases were classified as either high or low tumor *VEGFA* mRNA (n = 18 each) according to the median, log-rank test. (**c**) Association of baseline urinary VEGF-A levels with clinical response (n = 30). Cases were classified as either high or low urinary VEGF-A levels (n = 15 each) according to the median, Cochran–Armitage test. (**d**) Kaplan–Meier curves of progression-free survival (left) and overall survival (right) according to baseline urinary VEGF-A levels in dogs treated with sorafenib and piroxicam (n = 30). Cases were classified as either high or low urinary VEGF-A levels (n = 15 each) according to the median, log-rank test.

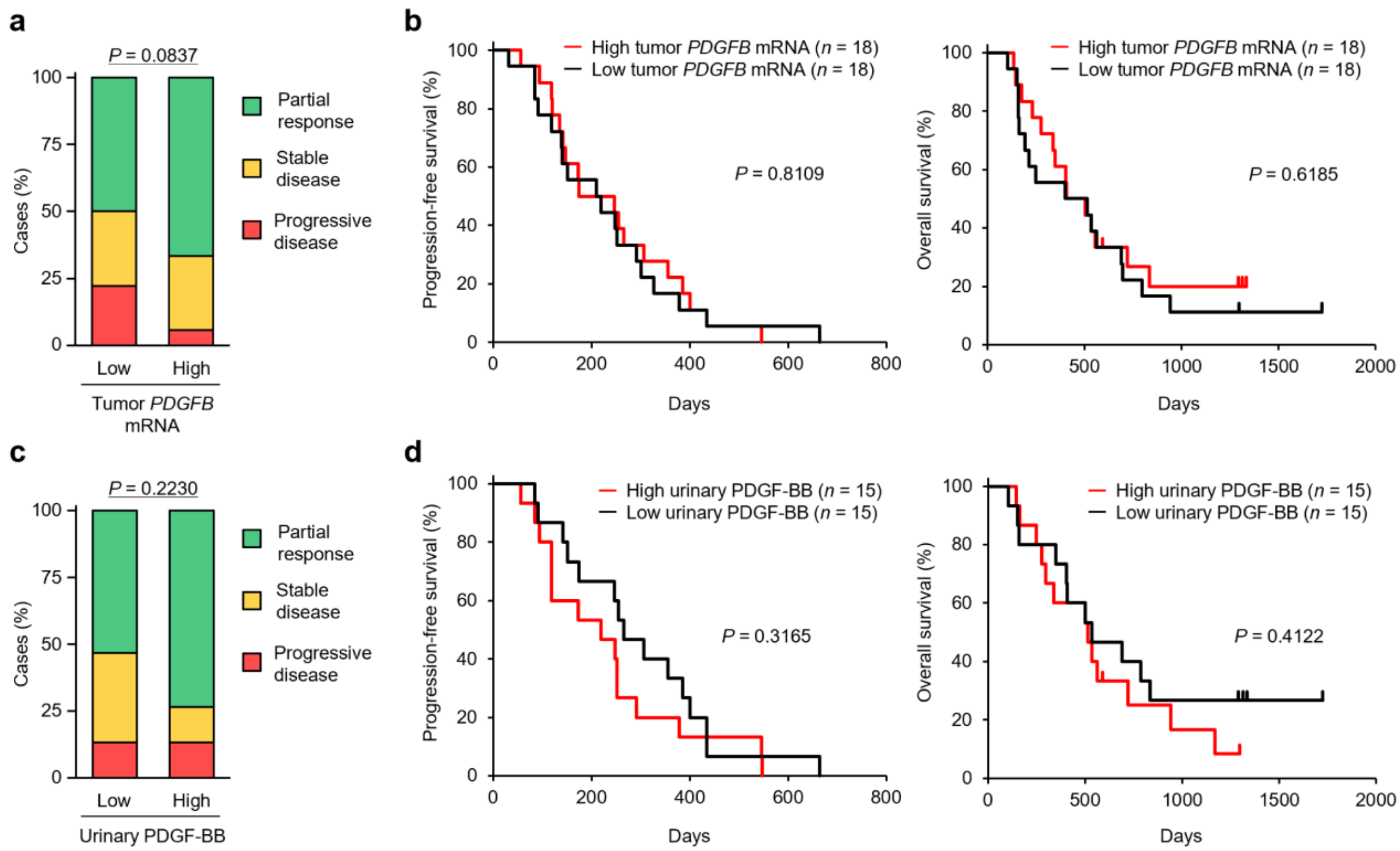

Figure S3. Association of PDGF expression levels with the clinical responses and survival in dogs treated with sorafenib and piroxicam. (**a**) Association of baseline tumor *PDGFB* mRNA expression with clinical response (*n* = 36). Cases were classified as either high or low tumor *PDGFB* mRNA (*n* = 18 each) according to the median, Cochran–Armitage test. (**b**) Kaplan–Meier curves of progression-free survival (left) and overall survival (right) according to baseline tumor *PDGFB* mRNA expression in dogs treated with sorafenib and piroxicam (*n* = 36). Cases were classified as either high or low tumor *PDGFB* mRNA (*n* = 18 each) according to the median, log-rank test. (**c**) Association of baseline urinary PDGF-BB levels with clinical response (*n* = 30). Cases were classified as either high or low urinary PDGF-BB levels (*n* = 15 each) according to the median, Cochran–Armitage test. (**d**) Kaplan–Meier curves of progression-free survival (left) and overall survival (right) according to baseline urinary PDGF-BB levels in dogs treated with sorafenib and piroxicam (*n* = 30). Cases were classified as either high or low urinary PDGF-BB levels (*n* = 15 each) according to the median, log-rank test.

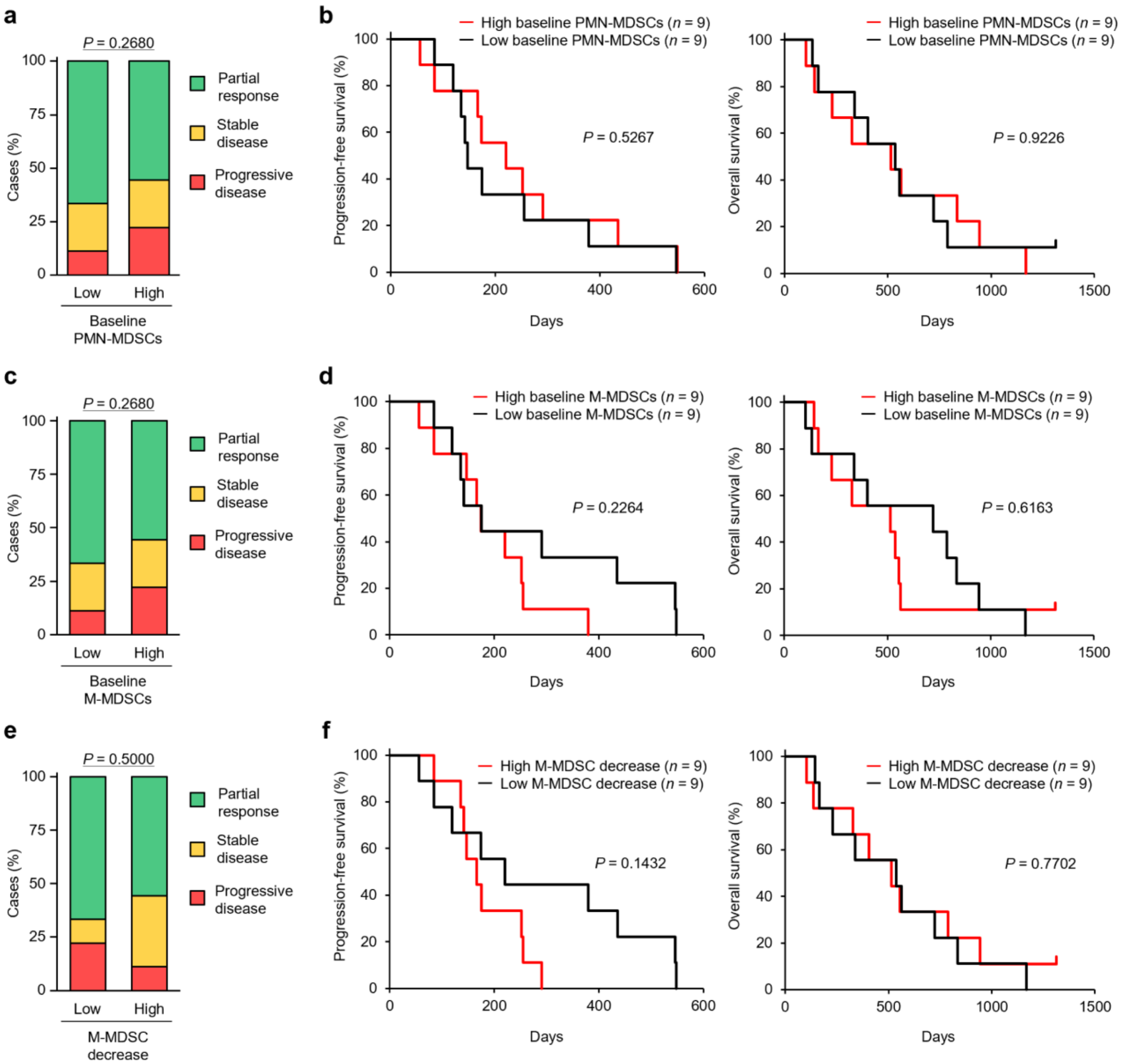

Figure S4. Association of myeloid-derived suppressor cells (MDSCs) with the clinical responses and survival in dogs treated with sorafenib and piroxicam. (**a**) Association between the baseline polymorphonuclear MDSCs (PMN-MDSCs) and clinical response (n = 18). Cases were classified as either high or low baseline PMN-MDSCs (n = 9 each) according to the median, Cochran–Armitage test. (**b**) Kaplan–Meier curves of progression-free survival (left) and overall survival (right) according to baseline PMN-MDSCs in dogs treated with sorafenib and piroxicam (n = 18). Cases were classified as either high or low baseline PMN-MDSCs (n = 9 each) according to the median, log-rank test. (**c**) Association between the baseline monocytic MDSCs (M-MDSCs) and clinical response (n = 18). Cases were classified as either high or low

baseline M-MDSCs (*n* = 9 each) according to the median, Cochran–Armitage test. (**d**) Kaplan–Meier curves of progression-free survival (left) and overall survival (right) according to baseline M-MDSCs in dogs treated with sorafenib and piroxicam (*n* = 18). Cases were classified as either high or low baseline M-MDSCs (*n* = 9 each) according to the median, log-rank test. (**e**) Association between the decrease in the ratio of M-MDSCs after sorafenib treatment and clinical response (*n* = 18). Cases were classified as either a high or low decrease in M-MDSCs (*n* = 9 each) according to the median, Cochran–Armitage test. (**f**) Kaplan–Meier curves of progression-free survival (left) and overall survival (right) according to the posttreatment reduction in M-MDSC in dogs treated with sorafenib and piroxicam (*n* = 18). Cases were classified as either a high or low decrease in M-MDSCs (*n* = 9 each) according to the median, log-rank test.
